# Commentary: Dietary index for gut microbiota and its inverse association with female infertility: evidence from NHANES 2013–2018

**DOI:** 10.3389/fnut.2025.1662189

**Published:** 2025-09-30

**Authors:** Rui Du, Binghui Jin, Tong Liu, Peng Zhang

**Affiliations:** The First Affiliated Hospital of Dalian Medical University, Dalian, China

**Keywords:** DI-GM, infertility, NHANES, dietary, dietary index for gut microbiota

## 1 Introduction

We read with interest the article by Cheng et al. entitled “Dietary Index for Gut Microbiota and Its Inverse Association with Female Infertility: Evidence from NHANES 2013–2018” (1). This cross-sectional study investigated the relationship between the dietary index for gut microbiota (DI-GM) and female infertility in the U.S. population using National Health and Nutrition Examination Survey (NHANES) 2013–2018 data. DI-GM is a novel metric that quantifies the relationship between diet and the diversity of gut microbiota. Per the authors' inclusion criteria, 3,008 participants were enrolled. Employing weighted linear regression and chi-square tests for group comparisons, the authors found that infertile patients had significantly lower DI-GM scores than women without infertility. Subsequent multivariate logistic regression and restricted cubic spline (RCS) analyses revealed a non-linear L-shaped inverse association between DI-GM and infertility risk. The subgroup analyses confirmed this relationship's robustness. This study comprehensively explored the relationship between DI-GM and female infertility, offering valuable evidence for nutritional interventions to improve reproductive health. However, we offer critical reflections below. This study comprehensively explored the relationship between DI-GM and female infertility, which provides valuable evidence for using nutrition to improve reproductive health. However, we have deeper reflections on this study.

## 2 Calculation of DI-GM

According to the description of the scoring criteria by Kase et al., the DI-GM comprises 14 dietary components: avocado, broccoli, chickpeas, coffee, cranberries, fermented dairy, fiber, green tea, soybean, and whole grains are classified as beneficial elements; red meat, processed meat, refined grains, and diets high in fat (≥40% of total energy intake) are identified as detrimental components (2). The Beneficial Gut Microbiota Score (BGMS) is calculated for beneficial components: a score of 1 is assigned if consumption ≥ the sex-specific median, otherwise 0. The Unfavorable Gut Microbiota Score (UGMS) is derived for detrimental components: a score of 0 is assigned if consumption ≥ the sex-specific median (or ≥40% for high-fat diet), otherwise 1. The DI-GM score is the sum of BGMS and UGMS. However, green tea was excluded from the final DI-GM calculation because NHANES 24-h dietary recall data does not capture specific tea consumption types (2). Consequently, the actual DI-GM score range is 0–13 (2). In this study, the authors state in the “2.2 Definition of DI-GM” section that the DI-GM score range is 0 to 14 points (1). It seems the authors may have overlooked Kase et al.'s description of green tea. We hope the authors can correct this mistake and recheck the validity of their conclusions to avoid misleading readers. Additionally, incorporating relevant discussions in the limitations section would help further enhance the study's rigor.

## 3 Adjustment for covariate

In this study, the authors selected several important covariates, including all demographic variables, pelvic infection, regular menstrual periods, female hormones taken, birth control pills, and so on (1). This is commendable. We noticed that the authors also adjusted for the history of hysterectomy and history of bilateral oophorectomy as covariates in model 3. However, we think these two covariates have some logical contradictions and could introduce statistical bias. Hysterectomy and bilateral oophorectomy are irreversible absolute causes of infertility, and the infertility status of these patients has nothing to do with factors like diet and microbiota (3). Putting surgical patients in the “infertility patient group” when their infertility mechanisms don't relate to the potential pathways of DI-GM might distort the real association. In another study on diet and infertility, Wang et al. excluded women who had undergone hysterectomy or bilateral oophorectomy from the study population (4). Therefore, to address the potential bias, we would recommend that the issue be explicitly acknowledged in the study's limitations section.

## 4 Conclusion

In conclusion, this study explores the relationship between DI-GM and female infertility among the U.S. population, offering new epidemiological evidence from a nutritional perspective for the prevention and treatment of infertility. However, we hope the authors will consider our suggestions to improve the study and offer more insights into female infertility in the future.
